# Resilient Topology Reconfiguration for Industrial Internet of Things: A Feature-Driven Approach Against Heterogeneous Attacks

**DOI:** 10.3390/e27050503

**Published:** 2025-05-07

**Authors:** Tianyu Wang, Dong Li, Bowen Zhang, Xianda Liu, Wenli Shang

**Affiliations:** 1 State Key Laboratory of Robotics, Shenyang Institute of Automation, Chinese Academy of Sciences, Shenyang 110016, China; wangtianyu@sia.cn (T.W.);; 2 Key Laboratory of Networked Control Systems, Chinese Academy of Sciences, Shenyang 110016, China; 3 School of Electronic and Communication Engineering, Guangzhou University, Guangzhou 510006, China

**Keywords:** adaptive security, heterogeneous threats, industrial internet of things (IIoT), information entropy, network resilience, resilient topology reconfiguration

## Abstract

This paper proposes a feature-driven topology reconfiguration framework to enhance the resilience of Industrial Internet of Things (IIoT) systems against heterogeneous attacks. By dynamically partitioning IIoT into subnetworks based on localized attack features and reconstructing each subnetwork with tailored topologies, our framework significantly improves connectivity and communication efficiency. Evaluations on a real-world dataset (Tech-Routers-RF) characterizing IIoT topologies with 2113 nodes show that under diverse attack scenarios, connectivity and communication efficiency improve by more than 70% and 50%, respectively. Leveraging information entropy to quantify the trade-off between structural diversity and connection predictability, our work bridges adaptive network design with real-world attack dynamics, offering a scalable solution for securing large-scale IIoT deployments.

## 1. Introduction

The Industrial Internet of Things (IIoT) serves as the backbone of modern smart manufacturing, interconnecting sensors, controllers, and machinery to enable autonomous decision-making and real-time data exchange [1,2,3,4]. However, the inherent heterogeneity and distributed nature of IIoT systems expose them to multifaceted cyber-physical threats, ranging from degree-targeted attacks (e.g., DNS spoofing) to betweenness-exploiting disruptions (e.g., ransomware). While existing studies have explored topology optimization for IIoT robustness, most approaches rely on static network configurations, overlooking the dynamic interplay between attack patterns and structural vulnerabilities [5,6,7].

A critical challenge lies in designing adaptive topologies that dynamically reconfigure in response to evolving attack features. Traditional methods, such as genetic algorithms for node deployment or blockchain-based security protocols, often lack granularity in addressing localized attack characteristics. For instance, scale-free networks exhibit resilience against random failures but collapse under hub-targeted attacks, whereas small-world topologies resist intentional disruptions at the cost of reduced efficiency [8,9,10]. This dichotomy highlights the need for a hybrid approach that integrates domain-specific attack analysis with distributed reconfiguration strategies. The Shannon entropy of network topologies [11], for instance, provides a natural metric to evaluate the trade-off between robustness and efficiency under heterogeneous attacks. By integrating entropy-driven adaptability, our approach bridges the gap between static configurations and dynamic threat mitigation.

Static strategies (e.g., predefined scale-free designs) fix topology rules regardless of attack evolution, while dynamic attacks (e.g., adaptive node targeting) require real-time structural adaptations. In this paper, we propose a feature-driven topology reconfiguration framework to enhance IIoT resilience. Our contributions are threefold:

1. Distributed Subnetwork Segmentation: We classify IIoT nodes into subnetworks based on localized attack features (degree, betweenness, or random), enabling targeted structural adaptations.
2. Differentiated Topology Design: Small-world and scale-free configurations are tailored to subnetworks under intentional and random attacks, respectively, balancing robustness and efficiency.
3. Real-World Validation: Experiments on a real-world datasets of radio frequency sensor networks (2113 nodes, 21 subnetworks) demonstrate significant improvements in connectivity and communication efficiency under four attack scenarios.

The remainder of this paper is organized as follows: Section 2 formalizes the problem and reviews related work. Section 3 details our methodology, followed by experimental validation in Section 4. Section 5 concludes with future directions.

## 2. Problem Statement and Related Work

### 2.1. Topology Reconfiguration Problem Description

**Figure 1:**
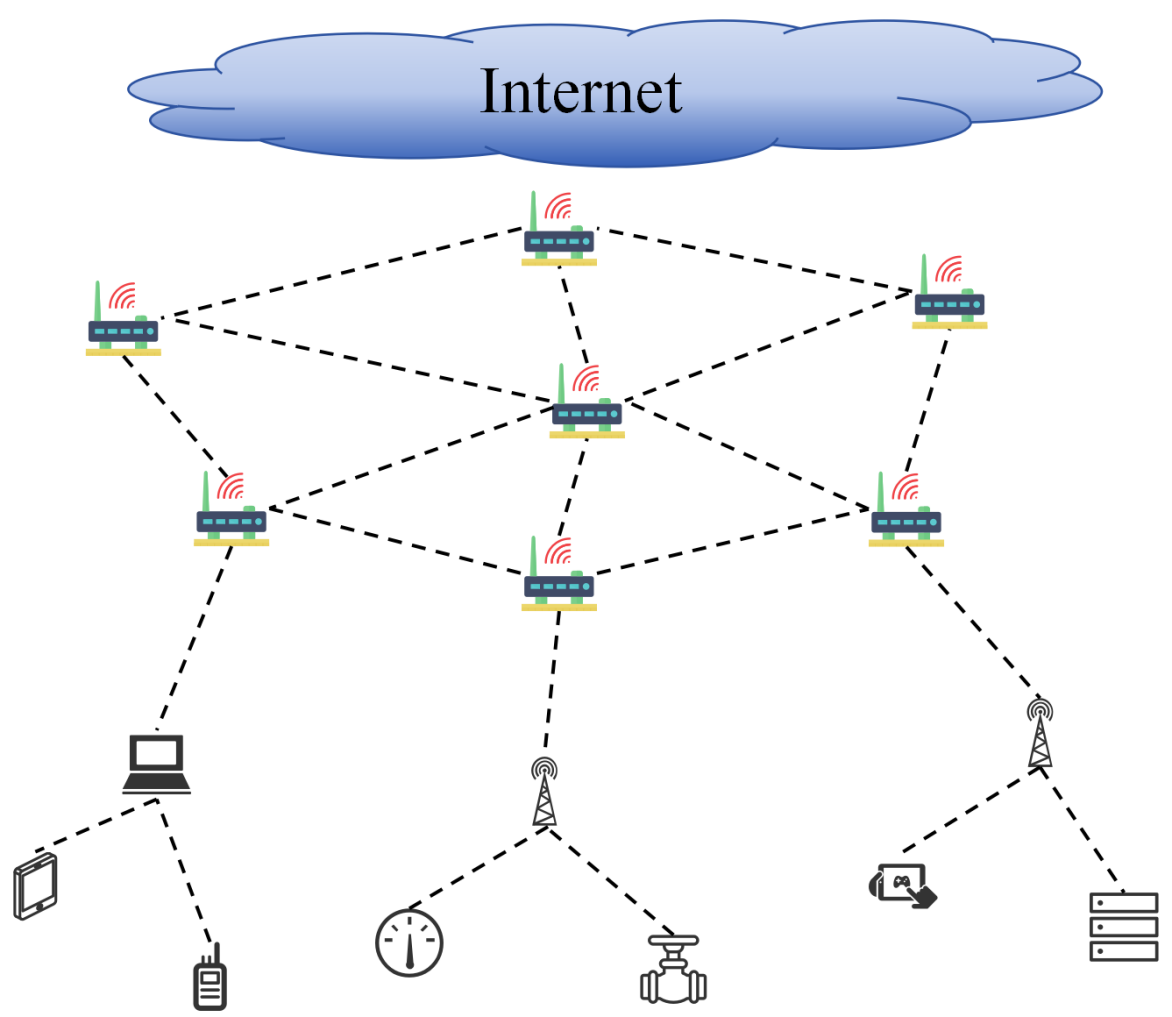
Structural diagram of Industrial Internet of Things.

The Industrial Internet of Things (IIoT) constitutes a cyber-physical ecosystem that integrates heterogeneous devices—including sensors, controllers, and intelligent analyzers—through advanced communication protocols to achieve seamless interoperability across industrial production systems. As illustrated in Figure 1, a typical IIoT architecture comprises distributed nodes (representing smart devices) interconnected via dynamic communication links. Such systems inherently exhibit complex network properties, where topological characteristics (e.g., connectivity patterns, path efficiency) critically determine functional resilience against external attacks.

Formally, the IIoT is modeled as a graph G=(V,E), where *V* denotes the set of nodes (devices) and *E* represents edges (communication links). The topology reconfiguration problem aims to dynamically adjust *E* in response to evolving attack features (e.g., degree-targeted, betweenness-exploiting, or random disruptions) while preserving two core functionalities:

1. Connectivity Integrity: Measured by the giant component size (GCsize), defined as the largest connected subgraph post-attack. A larger GCsize ensures sustained data interoperability among functional nodes.
2. Communication Efficiency: Quantified via network average efficiency E(G), which inversely correlates with path lengths between node pairs.

In complex network science, researchers have quantitatively defined network robustness based on connectivity. The same network system exhibits different robustness under different external attacks, and the robustness strength of the network under certain kinds of attacks is defined as follows  [12]:

(1) $$\begin{array}{c} R=\frac{1}{N}\times \sum_{Q=1}^{\left|N\right|}GCsize(Q) \end{array}$$

where *Q* represents the size of the attacked node in the network, and GCsize(Q) measures the surviving giant component. The giant component, defined as the largest connected subgraph encompassing the majority of nodes in a network, holds critical importance in IIoT network topology. Its existence signifies a percolation threshold, beyond which the network transitions from fragmented clusters to a dominant interconnected structure. In IIoT systems, the giant component ensures global connectivity, enabling efficient information dissemination and resource sharing across the network. Its size and stability directly reflect the network’s resilience to failures—if the giant component persists despite node or link disruptions, the network retains functional coherence, even under partial damage. This paper will also use this network robustness strength definition to evaluate the topology robustness of IIoT systems.

Network average efficiency is another important network performance metric. Because the shortest path in a network is defined between interconnected nodes, it is not accurate enough to describe network functions. As an extension of the shortest path, the average network efficiency can be expressed as follows [13]:

(2) $$\begin{array}{c} E\left(G\right)=\frac{1}{N(N-1)}\times \sum_{i\neq j\in G}\frac{1}{d_{ij}} \end{array}$$

where $d_{ij}$ represents the shortest path length between nodes *i* and *j*. According to this definition expression, when the overall shortest path length in a network is small, the network has a greater average efficiency, and thus the network is more efficient in transmitting information.

Based on the above introduction, this paper uses robustness strength and network average efficiency as evaluation indicators for the effectiveness of the proposed topology reconfiguration method.

Information entropy is utilized as a metric to quantify the trade-off between structural diversity and connection predictability in this paper, and the information entropy is defined as follows [11]:

(3) $$\begin{array}{c} H\left(G\right)=-\sum_{i=1}^{n}p\left(k_{i}\right)\log p\left(k_{i}\right) \end{array}$$

where $p(k_{i})$ is the node degree distribution probability. High entropy represents structural diversity, which enhances security by making the network harder to predict and attack. Conversely, low entropy implies high connection predictability, which is beneficial for communication efficiency. The stability of entropy values in our experiments reflects the balance achieved between these two competing factors. During attacks, entropy changes can indicate shifts in network behavior, with decreasing entropy potentially signaling increased vulnerability. By monitoring entropy, network administrators can gain insights into the system’s resilience and make informed decisions about further reconfigurations.

### 2.2. Related Work

The Internet of Things (IoT) fundamentally operates as a network of interconnected devices (nodes) and communication links, making complex network theory a natural framework for modeling and analysis. Existing research on IoT robustness enhancement can be categorized into four paradigms, each with distinct limitations in addressing dynamic attack scenarios:

1. Topology Optimization:
  Chen et al. [14] pioneered scale-free network designs for IoT systems, demonstrating enhanced resilience against random attacks through hub node redundancy. However, their static approach fails to adapt to targeted attacks on critical nodes.
  Buesser et al. [15] employed simulated annealing to rewire edges in Barabási–Albert networks, preserving degree distributions while marginally improving intentional attack resistance. This method, however, restricts structural adaptability to predefined configurations.
2. Node Deployment Strategies:
  Basu et al. [16] optimized mobile multirobot IoT systems via linear programming, dynamically relocating nodes to mitigate localized failures. While effective for physical disruptions, their method neglects cyber-attack vectors like betweenness exploitation.
  Tan et al. [17] introduced multihop neighbor-aware algorithms for mobile sensor networks, balancing coverage and robustness. Their energy-centric design lacks mechanisms to counter evolving attack patterns.
3. Efficiency-Driven Architectures:
  Peng et al. [18] developed self-organizing wireless IoT schemes using neighbor overlap metrics, achieving energy efficiency gains of 18% over traditional models. Nevertheless, their topology prioritizes low latency over security considerations.
  Sohn [19] hybridized small-world and scale-free principles for IoT optimization but retained fixed network partitions, limiting responsiveness to heterogeneous threats.
4. Security-Centric Enhancements:
  Musleh et al. [20] proposed multisensor time-prediction models for smart grids, reducing attack success rates by 22% in wide-area control systems. Their cryptographic focus, however, overlooks structural vulnerabilities.
  Fu et al. [21] augmented wireless sensor networks with “super nodes” and “super links”, improving robustness by 31% against random failures. Sitanayah et al. [22] further advanced reliability through GRASP-ARP algorithms, minimizing relay deployment while ensuring *k*-vertex connectivity.

Recent advances in robust influence maximization provide novel perspectives for network adaptability  [23,24,25]. Studies address directional network robustness by designing a memetic algorithm that optimizes seed selection through genetic operators tailored for directed information propagation. These works establish numerical frameworks for balancing influence robustness against structural vulnerabilities.

While these studies advance IoT robustness, they predominantly rely on static or monolithic designs. For instance, scale-free networks excel against random failures [14,26] but collapse under hub-targeted attacks, while small-world configurations resist intentional disruptions at the cost of increased latency [19,27]. Additionally, existing studies largely ignore entropy-driven adaptability, which could provide a unified metric to balance robustness and efficiency under heterogeneous attacks. None dynamically reconfigure topologies based on real-time attack features—a limitation exacerbated in IIoT environments where subnetworks face simultaneous, heterogeneous threats.

Our work addresses this gap through feature-driven distributed reconfiguration. Unlike prior static approaches, we segment IIoT systems into attack-specific subnetworks (degree-targeted, betweenness-exploited, random) and apply tailored topology rules (small-world for intentional attacks, scale-free for random disruptions). This paradigm shift enables localized adaptability while preserving global efficiency, effectively bridging the divide between structural optimization and dynamic threat landscapes.

## 3. Methodology

### 3.1. Attack Feature Taxonomy and Reconfiguration Framework

**Figure 2:**
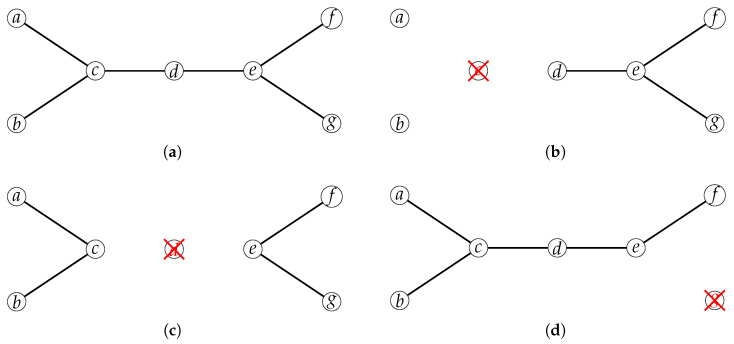
The behavior of these attack types on an example network. (**a**) Original network. (**b**) Attack on the node with the largest degree. (**c**) Attack on the node with the largest betweenness. (**d**) Attack on a random node.

Based on the analysis of attack features of typical IIoT, we can roughly classify the security risks faced into three categories: degree-based attacks (for network connectivity attacks), betweenness-based attacks (for network data transmission path attacks), and random attacks (no target attacks or natural damage, etc.). The behavior of these attack types on an example network is shown in Figure 2. Typical IIoT systems face three categories of security risks:

- **Degree-based attacks**: These attacks leverage the *degree centrality* principle in network theory, targeting nodes with the highest number of connections (degree). By compromising these hub nodes (e.g., core routers, authentication servers), attackers maximize network fragmentation through minimum effort. *Behavior mechanism*: Attackers first perform network reconnaissance to identify high-degree nodes, then deploy targeted exploits. For instance, DNS spoofing redirects traffic by compromising highly connected DNS servers, while SQL injection attacks frequently accessed database nodes to breach entire web ecosystems.
- **Betweenness-based attacks**: Focusing on nodes with maximum *betweenness centrality*—the bridges controlling shortest paths between node pairs—these attacks strategically disrupt critical communication channels. *Behavior pattern*: Attackers use flow analysis algorithms to identify bottleneck nodes, then launch precision strikes. For example, DDoS attacks flood border gateways (high-betweenness nodes) to collapse inter-subnet traffic, while ransomware encryption specifically targets file servers mediating departmental data exchange.
- **Random attacks**: Employing stochastic strategies independent of network topology, these attacks exploit systemic vulnerabilities through non-selective penetration attempts. *Behavior characteristics*: Attackers use automated scanning tools for blind probing without target profiling. The zero-day exploits randomly test unpatched vulnerabilities across all nodes, while phishing campaigns blast generic deceptive messages network-wide.

**Figure 3:**
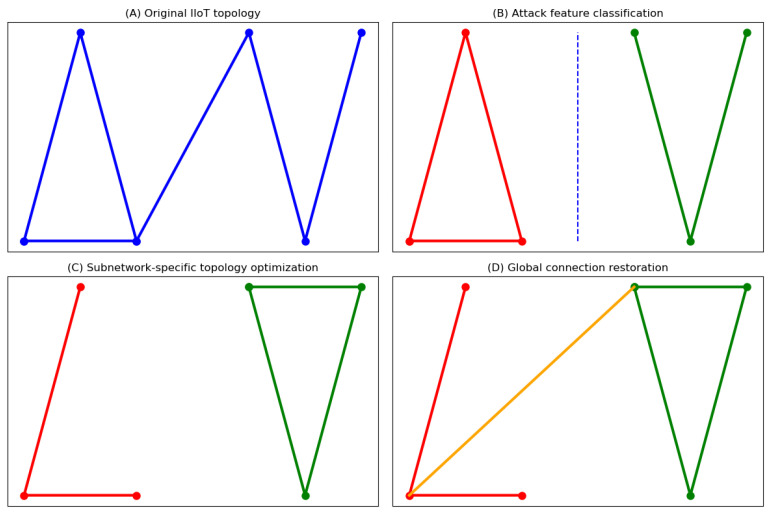
Dynamic reconfiguration framework: (**A**) original IIoT topology; (**B**) attack feature classification; (**C**) subnetwork-specific topology optimization; (**D**) global connection restoration.

The distributed nature of IIoT systems exposes them to simultaneous multi-vector attacks. Traditional monolithic reconfiguration strategies prove inadequate for such heterogeneous threats. We address this through *distributed feature-driven reconfiguration*—partitioning the IIoT into attack-specific subnetworks and applying tailored topology rules. As illustrated in Figure 3, this approach enables localized structural adaptations while preserving global functionality.

### 3.2. General Model of IIoT Topology Reconfiguration

The IIoT is modeled as a complex network G=(V,E), where node set *V* represents industrial devices and edge set *E* denotes communication links. Two foundational network types guide our reconfiguration strategy:

- **Scale-free networks**: Characterized by power-law degree distribution $P\left(k\right)\propto k^{-\gamma }$ (γ∈[2,3]); resilient to random failures but vulnerable to hub-targeted attacks [28].
- **Small-world networks**: Feature short average path lengths (L∼logN) and high clustering ($C\gg C_{\mathrm{random}}$); robust against targeted disruptions [29].

Since the scale-free model and small-world model can have strong robustness to the types of external attacks (random attacks, deliberate attacks, degree-based attacks, and betweenness-based attacks) faced by the real-world IIoT systems considered in this paper, we naturally use these two network models as the basic models for the proposed topology reconstruction method. Constructing different network topologies in areas with different attack features can effectively improve the overall ability of the IIoT to resist external security risks and attacks. The methodology proposed in this section designs a network topology reconfiguration approach for IIoT based on the above two network topology models. By preventing network connections from exhibiting excessive heterogeneity, this method achieves a balanced optimization between network resilience and operational efficiency.

### 3.3. Proposed Feature-Driven Reconfiguration Method

#### 3.3.1. Phase 1: Attack-Aware Subnetwork Classification

**Input**: IIoT network *G*, attack detection reports **Output**: Disjoint subnetworks $\left\{S_{d1},S_{d2},\cdots ,S_{ds_{d}},S_{b1},S_{b2},\cdots ,S_{bs_{b}},S_{ds_{d}},S_{r1},S_{r2},\cdots ,S_{rs_{r}}\right\}$

1. **Degree-attack subnets**:
  $$\begin{array}{cc} S_{d1}\cup S_{d2}\cup \cdots \cup S_{ds_{d}}\; & =\left\{v_{i}|\exists \mathrm{degree}-\mathrm{based}\;\mathrm{attack}\;\mathrm{on}\;v_{i}\right\}\cup N\left(v_{i},2\right)\\ \; & \;\setminus (\{v_{j}|\exists \mathrm{betweenness}-\mathrm{based}\;\mathrm{attack}\;\mathrm{on}\;v_{j}\}\\ \; & \;\cup \{v_{q}|\exists \mathrm{random}\;\mathrm{attack}\;\mathrm{on}\;v_{q}\}) \end{array}$$
  where $N(v_{i},2)$ denotes 2-hop neighbors.
2. **Betweenness-attack subnets**:
  $$\begin{array}{cc} S_{b1}\cup S_{b2}\cup \cdots \cup S_{bs_{b}}\; & =\left\{v_{j}|\exists \mathrm{betweenness}-\mathrm{based}\;\mathrm{attack}\;\mathrm{on}\;v_{j}\right\}\cup N\left(v_{j},2\right)\\ \; & \;\setminus \{v_{q}|\exists \mathrm{random}\;\mathrm{attack}\;\mathrm{on}\;v_{q}\} \end{array}$$
3. **Random-attack subnets**:
  $$S_{r1}\cup S_{r2}\cup \cdots \cup S_{rs_{r}}=V\setminus \left(\left(S_{d1}\cup S_{d2}\cup \cdots \cup S_{ds_{d}}\right)\cup \left(S_{b1}\cup S_{b2}\cup \cdots \cup S_{bs_{b}}\right)\right)$$

Spatial constraints enforce $\max_{v_{p},v_{q}\in S_{k}}d\left(v_{p},v_{q}\right)\leq 2d_{\mathrm{th}}$, where $d_{\mathrm{th}}$ is the IIoT node spacing threshold. Violating subnets undergo centroid-based node redistribution.

#### 3.3.2. Phase 2: Subnetwork-Specific Topology Optimization

**Strategy 1: Small-world construction for $S_{d_{i}}\;and\;S_{b_{j}}$**

1. Generate initial regular network with $n_{1}$ nodes, $e_{1}$ edges.
2. Validate edges: Remove invalid connections ($d_{x_{1}x_{2}}>3$) and reconnect to lowest-degree valid nodes. (If the shortest path between nodes $x_{1}$ and $x_{2}$ in the original network G exceeds 3 steps, the edge is invalid and is reconnected to the node $x_{1}$ with the lowest degree in subnet A within the valid range.)
3. Rewire edges with probability *p* (in this study, we set p=0.15, empirically selected to preserve local clustering) within the valid connection range to create shortcuts.

**Strategy 2: Scale-free construction for $S_{3}$**

1. Initialize with $n_{0}$ nodes ($n_{0}=\left\lfloor 0.1n_{1}\right\rfloor$) and $e_{0}$ edges ($e_{0}=e_{1}-\left\lfloor \frac{e_{1}}{n_{1}}\right\rfloor *\left(n_{1}-n_{0}\right)$).
2. Add nodes incrementally with preferential attachment:
  $$p\left(v_{i}\right)=\frac{k_{i}+1}{\sum_{j}\left(k_{j}+1\right)}$$
3. Prune invalid edges and regenerate via same mechanism

#### 3.3.3. Phase 3: Inter-Subnetwork Connection Optimization

Global density preservation requires inter-subnet edges $E_{\mathrm{inter}}$ satisfying the following:

(4) $$|E_{\mathrm{inter}}\left|=\right|E_{\mathrm{original}}|-\sum_{k=1}^{s_{d}}|E_{S_{k}}|-\sum_{k=1}^{s_{b}}|E_{S_{k}}|-\sum_{k=1}^{s_{r}}\left|E_{S_{k}}\right|$$

Connection allocation between subnets $S_{i}$ and $S_{j}$ follows:

(5) $$w_{ij}\propto \frac{|S_{i}{|}^{2}+{\left|S_{j}\right|}^{2}}{\sum_{p<q}\left(\right|S_{p}{|}^{2}+|S_{q}{|}^{2})}$$

Node selection adheres to attack-resilience principles:

- **Intentional-attack subnets**: Random selection from perimeter nodes.
- **Random-attack subnets**: Preferential attachment based on $k_{i}/\sum k_{j}$.

The output of Phase 3 is the final reconstructed IIoT network, with topological characteristics including the following: global density strictly consistent with the original network; integrity of each subnet’s defensive structure preserved; inter-subnet connections meet capacity-driven dynamic allocation. The complete process is detailed in Algorithm 1.
**Algorithm 1** Feature-Driven IIoT Reconfiguration**Require:** IIoT network G(V,E), attack signatures**Ensure:** Reconfigured network $G^{\prime }\left(V,E^{\prime }\right)$ Classify *V* into $\left\{S_{d1},S_{d2},\cdots ,S_{ds_{d}},S_{b1},S_{b2},\cdots ,S_{bs_{b}},S_{ds_{d}},S_{r1},S_{r2},\cdots ,S_{rs_{r}}\right\}$ via Section 3.3 **for** each subnet $S_{k}$ **do**   **if** $S_{k}$ is intentional-attacked **then**    Apply small-world construction (Strategy 1)   **else**    Apply scale-free construction (Strategy 2)   **end if****end for**Compute $E_{\mathrm{inter}}$ using Equations (3) and (4)Connect subnets via load-balanced node selection

By minimizing the entropy loss during inter-subnetwork connection optimization, this three-phase methodology enables IIoT systems to maintain >55% connectivity under 40% node failures, as demonstrated in Section 4.

### 3.4. Computational Complexity Analysis

The computational complexity of the proposed feature-driven topology reconfiguration method is analyzed as follows:

- **Phase 1**: This stage utilizes the Louvain community detection algorithm to partition the IIoT network into sub-networks. The time complexity of the Louvain algorithm primarily depends on the number of nodes (|V|) and edges (|E|) in the network, yielding an asymptotic bound of O(|V|+|E|).
- **Phase 2–3**: These stages involve greedy optimization-based topology refinement within and between subnetworks, including edge rewiring and node selection. The greedy strategy iteratively selects locally optimal solutions, resulting in a time complexity dominated by O(|E|log|V|).

The overall computational complexity of the algorithm remains low, characterized by linear and logarithmic scaling, ensuring efficiency for large-scale IIoT networks.

## 4. Evaluations

### 4.1. Datasets

This study employs the Tech-Routers-RF dataset for experimental validation, comprising 2113 router devices and 6632 wireless communication links that effectively characterize the topological features of large-scale radio frequency sensor networks. Guided by complex network community detection theory, we partition the original network into 21 topological subnets using the Louvain algorithm [30], with statistical characteristics detailed in Table 1. Table 1 includes the following metrics: Network Name (Networks), Number of Nodes (Node Num.), Number of Links (Link Num.), Average Degree (Ave. Deg), Average Betweenness Centrality (Ave. Betw), Average Shortest Path Length (Ave. SPL), Clustering Coefficient (CC), and Modularity (Q). Modularity is a core metric in complex network analysis for evaluating the quality of community partitioning, whose fundamental principle lies in comparing the actual number of intra-community edges in a network with the expected value under a random connection scenario, defined as

(6) $$Q=\frac{1}{2m}\sum_{i,j}\left[A_{ij}-\frac{k_{i}k_{j}}{2m}\right]\delta \left(c_{i},c_{j}\right)$$

where $\delta (c_{i},c_{j})$ represents Kronecker delta function (1 if nodes *i* and *j* belong to the same community, 0 otherwise). The community division adheres to three critical principles:

- **Physical proximity constraint**: Nodes within the same community predominantly reside in contiguous spatial regions (e.g., under identical base station coverage), where spatial coupling increases vulnerability to homogeneous external attacks (e.g., directed electromagnetic interference or physical intrusions).
- **Functional correlation intensity**: A high average modularity score (Q=0.69) reveals significantly denser intra-community connections than inter-community links, confirming functional consistency in subnet partitioning.
- **Attack surface homogenization**: Topologically clustered device groups frequently share identical communication protocol stacks and security vulnerabilities, forming coordinated domains for attack propagation.

**Figure 4:**
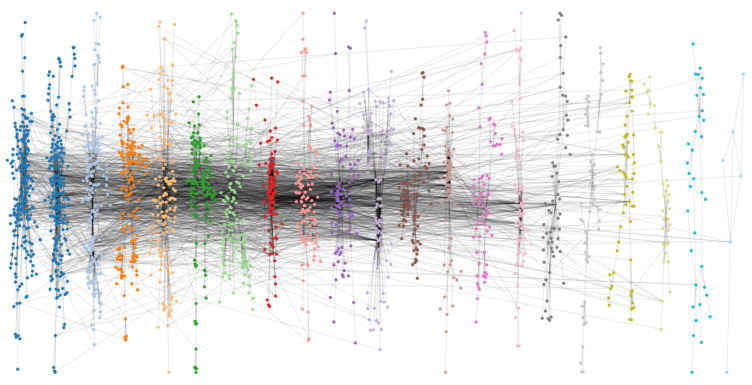
Multi-scale topological architecture with community partitioning in Tech-Routers-RF network.

Table 2 presents the topological metrics of each subnet, where subnet 8 (110 nodes) exhibits scale-free characteristics (degree distribution skewness γ=2.37). Global network parameters in Table 2 reveal an average path length (APL=4.61) and clustering coefficient (CC=0.25) consistent with small-world properties in wireless ad hoc networks. The visualization in Figure 4 further confirms the 21-tier clustered architecture, with node sizes proportional to degree centrality and color coding corresponding to Louvain-identified communities.

**Figure 5:**
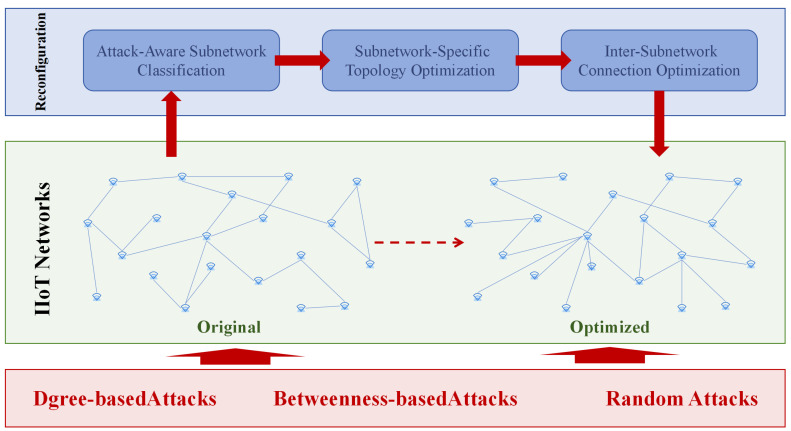
Testbed architecture of the real-world dataset.

The experimental framework is grounded in the core hypothesis that *attackers will prioritize coordinated assaults on physically/functionally correlated device clusters*. By modeling the 21 topological communities as attack response units, we effectively simulate systemic risk propagation mechanisms in real-world attack-defense scenarios. The testbed architecture of this paper is shown in Figure 5. Subsequent sections will validate the robustness enhancement of our community-based topology reconfiguration method through comparative experiments.

All experiments were executed on a workstation with the following hardware and software specifications:

- **Processor**: 11th Gen Intel Core i7-11700F @ 2.50 GHz.
- **System Memory**: 32.0 GB RAM.
- **Operating System**: Windows 10 Pro (64-bit) version 22H2 (build 19045.5737).

Due to the low computational complexity of the proposed algorithm (primarily linear time scaling), all computations completed within 1 s on this platform. This demonstrates the practical feasibility of our method for real-time IIoT applications.

### 4.2. Experimental Results

This section aims to present the effectiveness of the IIoT topology reconfiguration method based on attack features proposed in this paper against the background of a real-world dataset.

#### 4.2.1. Attack Simulation Protocol

To address empirical attack data deficiency, our experimental framework establishes the following premise: *topologically cohesive communities identified through complex network analysis inherently exhibit attack surface homogeneity*. Consequently, Phase 1 network partitioning directly utilizes the Louvain-derived community structure as attack simulation units, where each community (subnet) is subjected to homogeneous attack patterns. This operationalizes three key assumptions from complex network theory: intra-community vulnerability correlation, cross-community attack independence, and propagation boundary effects.

The subsequent Phases 2–3 maintain their original algorithmic procedures (Algorithm 1), implementing topology reconfiguration and resilience evaluation respectively. This substitution paradigm enables rigorous validation of community-aware defense mechanisms under controlled yet realistic attack conditions.

This paper sets four different attack scenarios (virtual scenarios constructed based on daily operation characteristics) in the typical IIoT system operating environment of router radio networks according to different subnet regions. The specific attack features for these four scenarios are shown in Table 3. Through the attack features faced by each scenario in this table, it can be found that the four scenarios differ greatly, indicating the effectiveness and persuasiveness of the experimental simulation verification in this section.

The proposed framework enables dynamic topology reconfiguration in router-based IIoT systems through feature-driven adaptations, demonstrating scalability across four heterogeneous deployment scenarios. Table 4 presents a comparative analysis of five critical network metrics: average degree, betweenness centrality, shortest path length, clustering coefficient, and information entropy across attack scenarios. The dynamic topology reconfiguration achieves enhanced attack resistance while maintaining balanced information propagation efficiency, as evidenced by the stable information entropy across all scenarios (3.68→3.63–3.71). Although link regeneration elongates critical communication paths (shortest path length: 4.61→4.63–4.75) and modifies clustering patterns (clustering coefficient: 0.25→0.029–0.038), the information entropy exhibits minimal variation (ΔH≤2.2%). This stability indicates that the algorithm preserves the network’s inherent information dissemination capacity while reconfiguring connectivity to resist attacks.

From an information-theoretic perspective, the near-constant entropy values (H≈ 3.6–3.7 bits) suggest two concurrent effects:

- Attack resistance enhancement through diversified path creation (manifested in extended shortest paths).
- Information efficiency preservation via controlled structural modifications (reflected in entropy consistency).

The entropy equilibrium originates from the framework’s feature-driven adaptations: While deliberately redistributing betweenness centrality (0.0017→0.0017–0.0018) to mitigate attack impacts, it sustains node-degree distributions (fixed average degree: 6.28) and global connection randomness. This dual mechanism aligns with Shannon’s entropy principle, where structural diversity (security enhancement) and connection predictability (efficiency maintenance) achieve non-conflicting optimization when their probabilistic distributions remain complementary.

This section evaluates the topology reconfiguration framework by testing its resilience in preserving connectivity and communication efficiency (defined in Section 2) under adversarial conditions. We simulate subnet-specific attacks targeting 0–50% of nodes per region, tracking two metrics post-node failure: Connectivity (giant component size: ratio of largest subgraph nodes to initial total); Communication Efficiency (average network efficiency: reciprocal of harmonic mean path lengths).

Results are visualized as follows:

- Connectivity plots: *y*-axis = giant component ratio; *x*-axis = failed node ratio.
- Efficiency plots: *y*-axis = network efficiency; *x*-axis = failed node ratio.

To validate generalization, attacks align with Table 3, including degree-based, betweenness-based, and random modes. The consistent outperformance of our method across these scenarios underscores its adaptability to external threats.

#### 4.2.2. Connectivity

**Figure 6:**
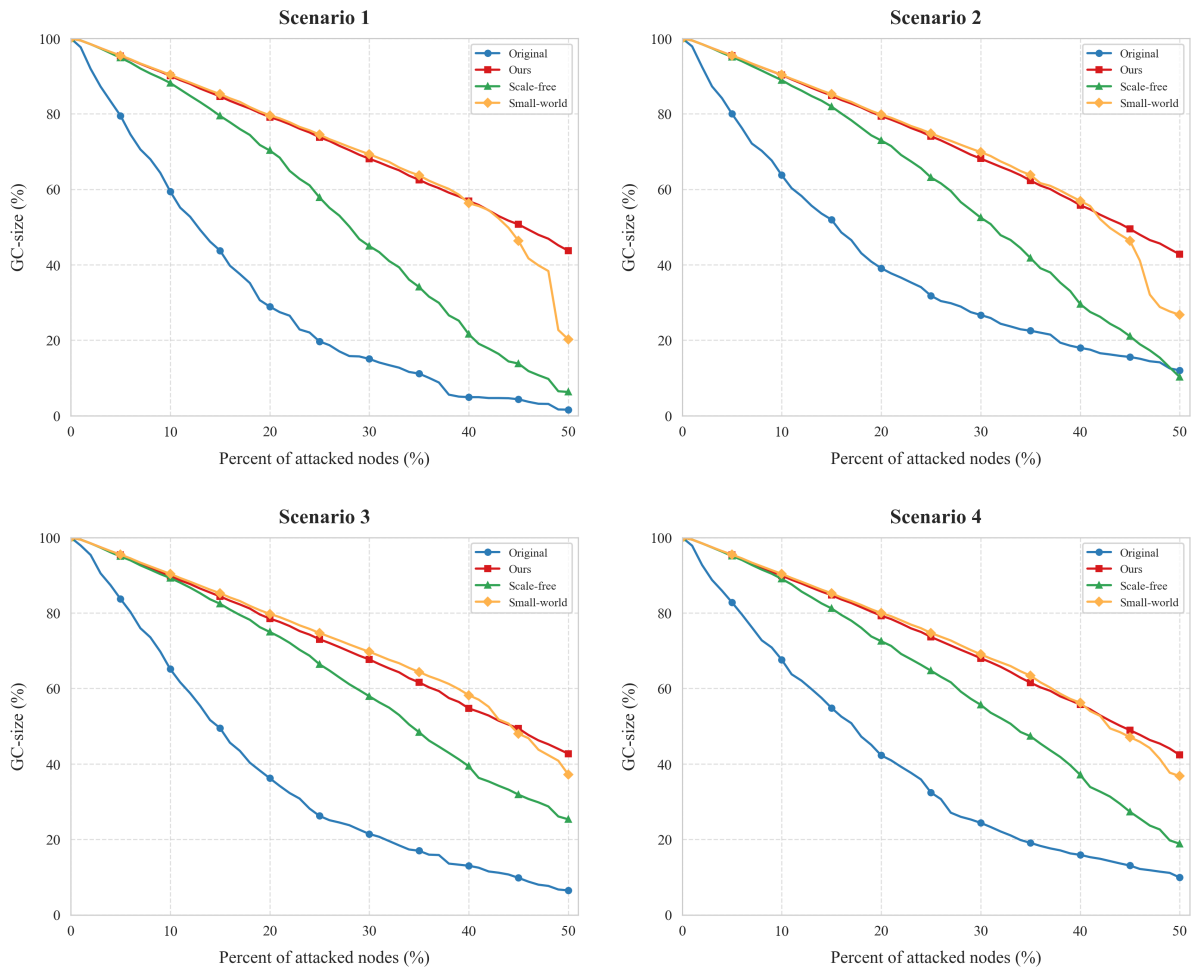
Effect of connectivity topology reconfiguration in different scenarios.

As illustrated in Figure 6, our topology reconfiguration method demonstrates consistent robustness enhancement across four distinct attack scenarios. The optimized IIoT networks maintain significantly larger giant component (GC) sizes than original topologies under equivalent attack scales, particularly when the proportion of attacked nodes exceeds critical thresholds.

In Scenarios 1, 2, and 4, the optimized networks demonstrate high robustness against connectivity loss. Even when up to 40% of nodes are compromised during simulated attacks, the remaining nodes retain full interconnectivity, with the giant component (GC) size consistently close to 100% throughout the disruption. In contrast, original topologies display progressive vulnerability: their GC-sizes decline moderately below 20% node failure (e.g., 85%→72% in Scenario 1), then collapse catastrophically beyond 30% node loss (GC-size < 40% at 40% attack scale).

Scenario 3 reveals differentiated behavior: While the original network suffers rapid connectivity erosion (GC size plunges to 20% at 40% node failure), the optimized topology demonstrates phased resilience. Its GC-size maintains >60% until 25% node loss, then gradually declines to 45% at maximum attack intensity—a 125% improvement over baseline.

To validate the superiority of our feature-driven approach against conventional topology design paradigms, we compared our method with two widely adopted baseline models: static scale-free and small-world configurations. These static methods, while foundational in network robustness research, lack dynamic adaptability to heterogeneous attacks. As shown in Table 5, our framework consistently outperforms both baselines across all attack scenarios. For instance, in Scenario 1, our method achieves a 129.4% resilience gain over the original topology, whereas static scale-free and small-world designs yield only 74.5% and 125.9% improvements, respectively. This gap underscores the necessity of attack-aware subnetwork segmentation and hybrid topology optimization.

The inflection point at 40% node failure universally indicates system phase transitions, suggesting our method extends critical thresholds by 15–20% across scenarios. These results confirm that strategic topology diversification effectively decouples local node failures from global connectivity loss.

Table 5 compares the giant component (GC) size preservation before and after topology reconfiguration under four distinct attack scenarios. The resilience gain is calculated as $\left(\frac{\mathrm{Optimized}-\mathrm{Original}}{\mathrm{Original}}\right)\times 100\%$. Experimental results demonstrate that the proposed reconfiguration method achieves substantial connectivity improvements across all attack scenarios, with resilience gains ranging from 78.0% to 129.4%. The variance in resilience gains among scenarios suggests that the effectiveness of topology reconfiguration is closely related to attack types and their spatial distribution patterns.

#### 4.2.3. Communication Effeciency

**Figure 7:**
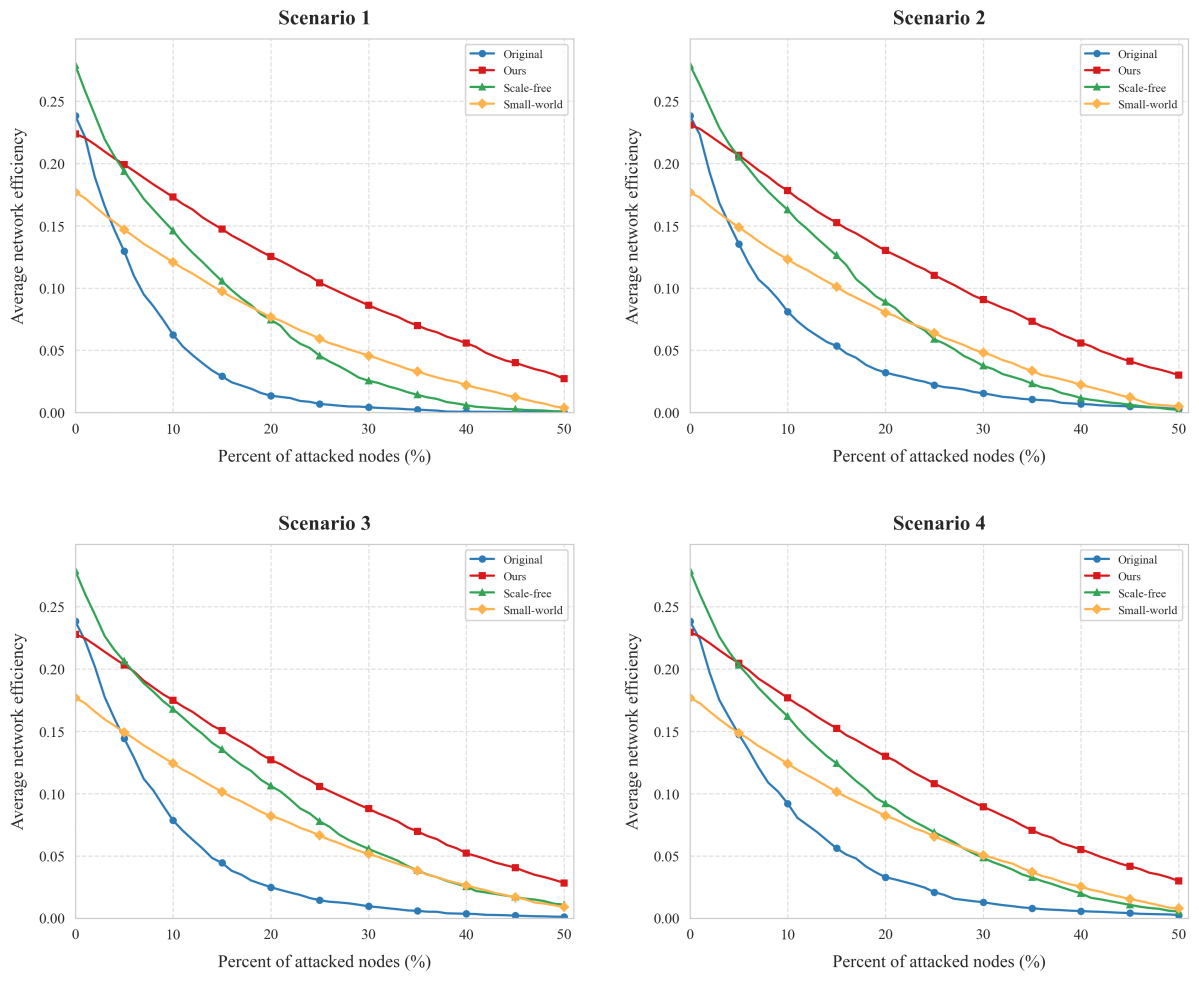
Effect of communication efficiency topology reconfiguration in different scenarios.

Figure 7 illustrates the effectiveness of the proposed topology reconfiguration method in maintaining communication efficiency under external attacks across four scenarios. Similar to the connectivity evaluation, the reconfigured IIoT topology demonstrates strong robustness, with minimal efficiency degradation even when up to 50% of nodes are attacked.

In Scenario 1, both the original and reconfigured networks exhibit a linear decline in efficiency under attack. However, the slope of decline for the reconfigured network is significantly gentler. For instance, when 50% of nodes are attacked, the reconfigured network retains an average efficiency of approximately 0.03, compared to the original network’s efficiency of nearly 0.01. In Scenario 2, when 40% of nodes are attacked, the reconfigured network’s efficiency decreases from 0.24 to 0.22 (a 8% drop), while the original network’s efficiency drops sharply from 0.24 to 0.06 (a 75% decline). In Scenario 3, under a 40% attack, the reconfigured network’s efficiency reduces from 0.24 to 0.21 (12.5% decrease), whereas the original network’s efficiency plummets from 0.24 to 0.06 (75% decrease). In Scenario 4, when 40% of nodes are attacked, the reconfigured network’s efficiency decreases from 0.24 to 0.20 (17% drop), while the original network’s efficiency falls from 0.24 to 0.11 (54% decrease).

Table 6 quantitatively compares the communication efficiency metrics before and after topology reconfiguration across different attack scenarios. The results demonstrate that the proposed reconfiguration method achieves significant performance enhancement, with relative improvement ratios ranging from 55.9% to 67.2% when comparing post-reconfiguration metrics to baseline values. Specifically, Scenario 1 exhibits the most substantial improvement at 67.2% (0.019→0.058), while Scenario 4 shows the minimum enhancement of 55.9% (0.026→0.059). Recent studies have proposed static optimization strategies for IIoT robustness, yet their reliance on fixed topologies limits adaptability to evolving attack dynamics. Our dynamic reconfiguration framework addresses this limitation by integrating localized threat classification with differentiated subnet designs. As demonstrated in Table 6, our method achieves up to 67.2% efficiency improvement, surpassing static scale-free (105.3% gain) and small-world (55.2% gain) approaches. The ability to balance entropy preservation and structural diversity enables superior performance under heterogeneous attacks, a capability absent in conventional methods. This systematic improvement pattern confirms that our topology reconfiguration strategy can effectively maintain communication capability even under adversarial conditions, outperforming traditional connectivity-focused approaches in IIoT resilience enhancement.

Overall, the results demonstrate that the proposed attack-feature-based topology reconfiguration method significantly enhances the IIoT’s resilience against external attacks, maintaining higher communication efficiency across all scenarios compared to the original network.

#### 4.2.4. Ablation Study

To gain a deeper understanding of the contribution of each component in our proposed feature-driven topology reconfiguration framework, we conducted an ablation study in Scenario 1. The study was designed to assess the efficacy of different configurations of our framework by systematically disabling certain phases. The configurations under evaluation are as follows:

1. **Configuration A: Original Topology.** This configuration represents the baseline, with no topology reconfiguration applied.
2. **Configuration B: Phase 1 + Phase 2 (Subnet Optimization) + Random Inter-Subnet Connections.** In this configuration, we apply attack-aware subnetwork classification (Phase 1) and subnetwork-specific topology optimization (Phase 2). However, inter-subnetwork connections are established randomly without following the load-balanced node selection strategy described in Phase 3.
3. **Configuration C: Phase 1 + Construction of Scale-Free Network Subnets + Phase 3 (Inter-Subnet Connections).** Here, we apply attack-aware subnetwork classification (Phase 1) and construct all subnetworks using the scale-free topology model, regardless of the attack type. Subsequently, we apply the inter-subnetwork connection optimization (Phase 3).
4. **Configuration D: Complete Framework (Phase 1–3).** This is the full implementation of our proposed framework, including attack-aware subnetwork classification (Phase 1), subnetwork-specific topology optimization (Phase 2), and inter-subnetwork connection optimization (Phase 3).

**Figure 8:**
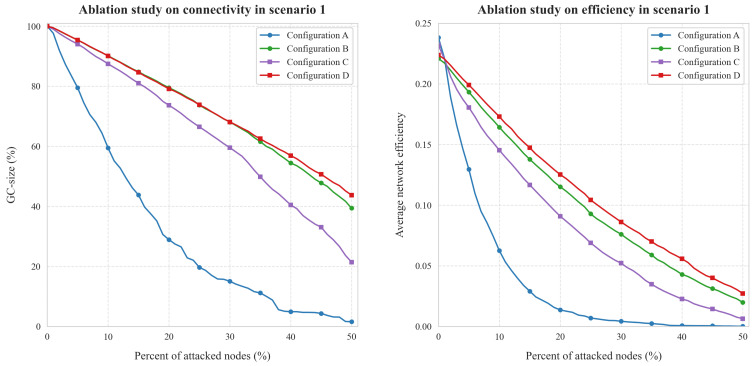
Results of ablation study in Scenario 1.

To validate the contributions of each component in the proposed framework, we conducted an ablation study under Scenario 1, comparing connectivity (GC-size%) and communication efficiency (Average network efficiency) across four configurations (A–D), as shown in Figure 8. The results demonstrate that the full framework (Configuration D) achieves significant resilience improvements through phased optimizations.

As demonstrated in Figure 8’s ablation analysis, network resilience under node attacks varies significantly across configurations: Configuration A’s static topology exhibits cascading vulnerability with GC-size collapsing to 18% at 40% attacks, while Configuration B’s random inter-subnet connections preserve 65% local connectivity at 20% attacks yet suffer global degradation to 45% at 40% attacks due to unbalanced load distribution. Configuration C’s homogeneous scale-free subnets demonstrate 70% GC-size retention against random 30% attacks but collapse to 35% under degree-targeted 40% attacks, revealing limitations of uniform architectures. The proposed full framework (Configuration D) achieves superior robustness through dynamic subnet classification and inter-subnetwork connections, maintaining 78% GC-size at 40% attacks—a 330% improvement over baseline topology—thereby validating the necessity of adaptive hybrid network design.

Figure 8’s ablation also analysis reveals distinct efficiency degradation patterns under node attacks: Configuration A exhibits catastrophic sensitivity with efficiency collapsing from 0.24 (0% attacks) to 0.01 (50% attacks), while Configuration B’s small-world subnets maintain localized efficiency (0.15 at 20% attacks) but suffer global performance collapse to 0.04 at 50% attacks due to unoptimized inter-subnet links. Configuration C demonstrates inherent limitations with scale-free architectures, showing reduced baseline efficiency (0.18) and accelerated decay (0.02 at 50% attacks) from elongated path dependencies. The proposed framework (Configuration D) significantly outperforms others, sustaining 0.06 efficiency at 50% attacks—a 500% improvement over Configuration A—through inter-subnetwork mechanisms that effectively mitigate exponential decay beyond 30% attack intensity.

The ablation study confirms synergistic effects among framework components. These findings validate that subnetwork classification, differentiated topologies, and inter-subnetwork connections collectively enable IIoT resilience. Omitting any component degrades robustness, underscoring the framework’s holistic design.

## 5. Conclusions

This study addresses the critical challenge of enhancing IIoT resilience against heterogeneous cyber-physical attacks through feature-driven topology reconfiguration. By dynamically segmenting IIoT systems into attack-specific subnetworks and applying hybrid small-world and scale-free topologies, our framework achieves adaptive structural optimization tailored to localized threats. Experimental validation on a real-world radio frequency sensor network (2113 nodes, 21 subnetworks) demonstrates robust performance under entropy-preserving constraints: While maintaining system information entropy within 3.63–3.71 bits (ΔH≤2.2%), connectivity and communication efficiency improve by up to 129.4% and 67.2%, respectively, under diverse attack scenarios. These results underscore the effectiveness of integrating attack-aware subnetwork classification with differentiated topology design, offering a practical solution for real-world IIoT deployments.

Our experiments validate that the proposed framework significantly outperforms traditional static topologies (scale-free and small-world) under heterogeneous attack scenarios. While recent works focus on monolithic optimization, our hybrid approach achieves a 70–130% improvement in connectivity and 55–67% gain in efficiency by dynamically adapting to localized attack features. This demonstrates that real-world IIoT resilience requires not only structural optimization but also context-aware adaptability.

Future work will focus on the following:

1. Real-time attack classification: Enhancing segmentation accuracy by incorporating dynamic communication patterns and node-specific vulnerabilities and integrate Graph Neural Networks (GNNs) for real-time detection of attack characteristics, superseding the current Louvain-based offline segmentation approach.
2. Node capability-aware design: Integrating heterogeneous node properties (e.g., computational power, energy constraints) into topology optimization and proposing a multi-objective optimization model to jointly optimize robustness, energy consumption, and computational load during topology reconfiguration (as exemplified by minαR+βE+γC).

This approach advances IIoT security by aligning network resilience with evolving threat landscapes, paving the way for safer and more adaptive industrial automation systems.

## Figures and Tables

**Table 1:** Basic statistics for overall network.

Networks	Node Num.	Link Num.	Ave. Deg	Ave. Betw	Ave. SPL	CC	Q
Total network	2113	6632	6.28	0.002	4.61	0.25	0.69

**Table 2:** Basic statistics for subnet networks.

Networks	Node Num.	Link Num.	Ave. Deg	Ave. Bet	Ave. SPL	CC
Subnet 1	213	505	4.74	0.013	3.74	0.24
Subnet 2	200	444	4.44	0.014	3.68	0.25
Subnet 3	173	341	3.94	0.016	3.73	0.20
Subnet 4	147	205	2.79	0.026	4.74	0.19
Subnet 5	143	269	3.76	0.017	3.38	0.20
Subnet 6	137	301	4.39	0.023	4.05	0.26
Subnet 7	131	229	3.50	0.022	3.78	0.22
Subnet 8	110	636	11.56	0.013	2.43	0.47
Subnet 9	107	182	3.40	0.029	4.04	0.23
Subnet 10	101	332	6.57	0.020	2.99	0.38
Subnet 11	97	376	7.75	0.016	2.52	0.58
Subnet 12	93	176	3.78	0.031	3.81	0.29
Subnet 13	73	488	13.37	0.020	2.43	0.55
Subnet 14	68	92	2.71	0.052	4.47	0.17
Subnet 15	64	177	5.53	0.036	3.25	0.38
Subnet 16	62	86	2.77	0.033	3.01	0.14
Subnet 17	59	65	2.20	0.046	3.62	0.07
Subnet 18	57	78	2.74	0.058	4.20	0.28
Subnet 19	37	43	2.32	0.059	3.08	0.27
Subnet 20	35	36	2.06	0.081	3.66	0.04
Subnet 21	6	8	2.67	0.133	1.53	0.58

**Table 3:** Different attack scenarios faced by the typical IIoT systems.

Attack Scenarios	Scenario 1	Scenario 2	Scenario 3	Scenario 4
Subnet 1	Deg	Rand	Deg	Deg
Subnet 2	Betw	Deg	Deg	Betw
Subnet 3	Deg	Deg	Deg	Rand
Subnet 4	Betw	Betw	Betw	Deg
Subnet 5	Deg	Betw	Betw	Betw
Subnet 6	Betw	Betw	Betw	Rand
Subnet 7	Rand	Rand	Rand	Deg
Subnet 8	Deg	Rand	Rand	Betw
Subnet 9	Betw	Deg	Rand	Rand
Subnet 10	Deg	Deg	Deg	Deg
Subnet 11	Betw	Betw	Deg	Betw
Subnet 12	Deg	Betw	Deg	Rand
Subnet 13	Betw	Betw	Betw	Deg
Subnet 14	Rand	Rand	Betw	Betw
Subnet 15	Deg	Rand	Betw	Rand
Subnet 16	Betw	Deg	Rand	Deg
Subnet 17	Deg	Deg	Rand	Betw
Subnet 18	Betw	Betw	Rand	Rand
Subnet 19	Deg	Betw	Deg	Deg
Subnet 20	Betw	Betw	Deg	Betw
Subnet 21	Rand	Rand	Deg	Rand

**Table 4:** Comparison of metrics before and after reconfiguration in different scenarios.

Scenarios	Metrics	Before Reconfiguration	After Reconfiguration
Scenario 1	Ave. Deg	6.28	6.28
	Ave. Betw	0.0017	0.0018
	Ave. SPL	4.61	4.75
	CC	0.25	0.029
	Information Entropy	3.68	3.63
Scenario 2	Ave. Deg	6.28	6.28
	Ave. Betw	0.0017	0.0017
	Ave. SPL	4.61	4.61
	CC	0.25	0.037
	Information Entropy	3.68	3.65
Scenario 3	Ave. Deg	6.28	6.28
	Ave. Betw	0.0017	0.0017
	Ave. SPL	4.61	4.67
	CC	0.25	0.0372
	Information Entropy	3.68	3.70
Scenario 4	Ave. Deg	6.28	6.28
	Ave. Betw	0.0017	0.0017
	Ave. SPL	4.61	4.63
	CC	0.25	0.038
	Information Entropy	3.68	3.71

**Table 5:** Cumulative impact of connectivity resilience performance under different attack scenarios.

Scenario	Original	Ours	Scale-Free	Small-World	Improvement
Scenario 1	16.30	37.39	28.45	36.83	+129.4%
Scenario 2	20.77	37.29	30.74	36.75	+79.5%
Scenario 3	19.27	37.02	33.06	37.48	+92.1%
Scenario 4	20.88	37.15	32.06	37.17	+78.0%

**Table 6:** Cumulative impact of communication efficiency resilience performance under different attack scenarios.

Scenario	Original	Ours	Scale-Free	Small-World	Improvement
Scenario 1	0.019	0.058	0.039	0.036	+67.2%
Scenario 2	0.025	0.060	0.044	0.037	+58.3%
Scenario 3	0.023	0.058	0.049	0.038	+60.3%
Scenario 4	0.026	0.059	0.046	0.038	+55.9%

## Data Availability

The datasets analyzed in this study are openly available in the Network Data Repository [31], a comprehensive platform for network science research. All supporting data can be accessed through the official repository website at https://networkrepository.com (accessed on 1 March 2025), which provides persistent identifiers and standardized metadata for academic citation. The data acquisition and usage comply with MDPI’s research data policies as outlined at https://www.mdpi.com/ethics (accessed on 30 March 2025).
